# Diversity of Viruses in *Ixodes ricinus* in Europe including Novel and Potential Arboviruses

**DOI:** 10.1155/2023/6661723

**Published:** 2023-11-21

**Authors:** Bianca Elena Bratuleanu, Cristian Răileanu, Amal Bennouna, Delphine Chretien, Thomas Bigot, Pablo Guardado-Calvo, Gheorghe Savuta, Sara Moutailler, Marc Eloit, Sarah Temmam

**Affiliations:** ^1^Pathogen Discovery Laboratory, Institut Pasteur, Paris, France; ^2^Regional Center of Advanced Research for Emerging Diseases, Zoonoses and Food Safety, “Ion Ionescu de la Brad” Iasi University of Life Sciences, Iași, Romania; ^3^WOAH Collaborating Centre for Detection and Identification in Humans of Emerging Animal Pathogens, Institut Pasteur, Paris, France; ^4^Bioinformatics and Biostatistics Hub, Institut Pasteur, Université Paris Cité, Paris, France; ^5^Institut Pasteur, Université Paris Cité, Structural Biology of Infectious Diseases Unit, F-75015, Paris, France; ^6^ANSES INRAE, Ecole Nationale Vétérinaire d'Alfort, UMR BIPAR, Laboratoire de Santé Animale, Maisons-Alfort, France; ^7^Ecole Nationale Vétérinaire d'Alfort, University of Paris-Est, Maisons-Alfort, France

## Abstract

Tick-borne pathogens are responsible for many vector-borne diseases in Europe, causing important problems for human and animal health. The composition of viral communities in ticks and their interactions with pathogens is little understood, especially in Eastern Europe, an area that represents a major hub for animal-arthropod vectors exchanges. In this study, we used metatranscriptomics to characterize the virome of 2,753 *Ixodes ricinus* ticks collected from France and Romania, focusing on viruses that could potentially have implications for human or animal health. Among the great viral diversity of viruses identified, we reported a novel strain of Tribec virus, an important human pathogen that was found in Romanian ticks. We detected viruses belonging to the *Phenuiviridae* and *Nairoviridae* families close to human and animal pathogens, suggesting that these viruses could constitute novel arboviruses. We used luciferase immunoprecipitation system targeting external viral proteins of viruses identified among the *Sedoreoviridae, Phenuiviridae*, and *Nairoviridae* families to screen serum samples from small ruminants' exposed to tick bites. The results suggest that part (approximately 12%, 95% CI 9.1–16.2) of the small ruminant population from Danube Delta, was exposed to viruses related to bi- or tri-segmented nairoviruses, but cross-reactive viruses could not be confirmed with certainly. The strategy developed in this study serves as a key step in predicting potential new disease outbreaks and can be readily adapted to study other reservoirs, vectors, and interfaces involving susceptible hosts.

## 1. Introduction

Ticks are obligate hematophagous arthropods of animals and humans that can transmit many pathogenic agents, including viruses, bacteria, and protozoa [1]. *Ixodes ricinus* is the most common and epidemiologically important species of ticks in Europe and has a major impact on animal and public health due to its ability to feed on various animal species and to the numerous pathogens it transmit [2]. Besides being the main vector for *Borrelia burgdorferi* sensu lato which causes the Lyme borreliosis, *I. ricinus* can also transmit other medically significant bacterial pathogens, such as *Neoehrlichia mikurensis*, *Anaplasma phagocytophilum*, *Rickettsia helvetica*, *Rickettsia monacensis*, *Coxiella burnetii*, and *Francisella tularensis*, or viral agents like tick-borne encephalitis or louping ill viruses [3, 4].

Compared to these extensively studied pathogens, research on tick-transmitted arboviruses and especially tick-borne orbiviruses is limited despite several reports of their importance for public health [5–7]. The *Sedoreoviridae* family consists of six genera, which have the ability to infect a diverse range of hosts such as mammals, birds, crustaceans, arthropods, algae, and plants [8]. Within the family, the *Orbivirus* genus is primarily associated with animal arboviruses that cause bluetongue disease in sheep, cattle, goats, and wild ungulates; African horse sickness in horses, donkeys, and dogs; and epizootic hemorrhagic deer fever [9]. Some orbiviruses are spread by insects (such as midges, flies, and mosquitoes) [10] and others by ticks, such as the Great Island serogroup [11]. Actually, the Great Island group includes Great Island virus (GIV), Tribec virus (TRBV), Kemerovo virus (KEMV), Lipovník virus (LIPV), Broadhaven virus (BRDV), Nugget virus (NUGV), and Muko virus (MUV) [12]. TRBV was first isolated from *I. ricinus* ticks from Tribec mountain range (Slovakia, 1966), and later from *Haemaphysalis punctata* (Romania) and other tick species originating from the Czech Republic, Belarus, Ukraine, Moldova, Russia (Volga), Italy, and Germany [7, 11, 13, 14], suggesting that the virus is circulating mostly among tick populations in Eastern Europe. For the vertebrate counterpart, TRBV was described in Slovakia and Czechoslovakia in rodents, birds, brown hares, wild rabbits, and small ruminants [13, 15, 16]. In humans, TRBV-specific antibodies were detected in patients presenting febrile illness and aseptic meningitis [17]. However, the human health and social impact of this virus are still unknown in Europe.

Due to the rapid development of next-generation sequencing during the past decade, a significant number of new viruses have been discovered in ticks around the world [18–26]. For example, orthonairoviruses transmitted by ticks are a serious threat to public health worldwide [27]. In addition to the well-known Crimean–Congo hemorrhagic fever virus, there are four emerging orthonairoviruses, yet identified only in China: Tacheng tick virus 1 [28], Songling virus [29], Beiji nairovirus [30], and Yezo virus [31], all associated to human febrile illnesses. Furthermore, a nairo-like bisegmented virus group was recently described, which includes Pustyn virus, South Bay virus, Norway nairovirus 1, Gakugsa tick virus, and Grotenhout virus [19, 32–35], but the relevance of these nairo-like viruses for public health remains to be unveiled, because some of them are actively circulating in tick populations of Eastern Europe. Similarly, within the *Phenuiviridae*, a novel phlebovirus named Tacheng tick virus 2 (TaTV2) has been detected in *Dermacentor marginatus* from China [36] and was also found in one patient's blood, indicating a possible vectorial transmission of TaTV2, which highlight the need to monitor the emergence of phleboviruses in humans in close contact with ticks [36].

In this study, we aimed to describe the virome diversity of *I. ricinus* ticks from France (Alsace and Ardennes regions) and Romania (Iasi and Tulcea regions), collected between 2010 and 2021. Among the huge diversity of viruses identified, including novel orthonairoviruses for which the spillover potential is unknown, we reported the discovery of one novel strain of Tribec virus identified in ticks from Danube Delta region, Romania. We conducted a comprehensive sero-epidemiological study in the Romanian small ruminant population in contact with ticks and demonstrated the low risk of vertebrate infection of these new viruses.

## 2. Materials and Methods

A number of 2,236 questing nymph and adult ticks were sampled from the environment, in Northeastern France, between 2010 (Alsace) and 2012 (Ardennes). The nymphs were grouped into pools of 15 specimens (116 pools) and adults were treated individually. Furthermore, 202 adult ticks (20 pools) were directly collected from sheep in Eastern Romania, Danube Delta region, during October 2020 and May 2021, as described previously [22]. Additionally, 315 adult ticks (39 pools) were collected using the flagging method between March and September 2015, in Iasi County, Romania. Finally, a total of 331 small ruminants were blood sampled in six different locations from Danube Delta Biosphere Reserve, Romania. The details of sampling, sequencing, and bioinformatics analysis are presented in the Supplementary Materials (Supplementary 1, Supplementary 2, and Supplementary 3).

## 3. Results

### 3.1. Diversity and Composition of the Virome of *Ixodes ricinus* in Europe

**Figure 1:**
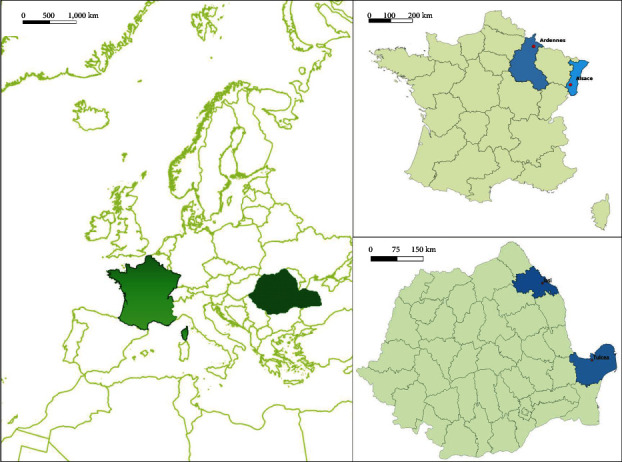
Sampling sites map of Romania and France.

In the present study, we conducted metatranscriptomics analysis of 2,753 *I. ricinus* ticks collected in France and Romania (Figure 1) from the environment and on small ruminants. The taxonomic assignation of sequences revealed that eukaryote and bacteria-related sequences were the most abundant in all libraries, ranging from 37% to 56%, and viruses represented between 10% and 15% of sequences, depending on the sample considered.

**Figure 2:**
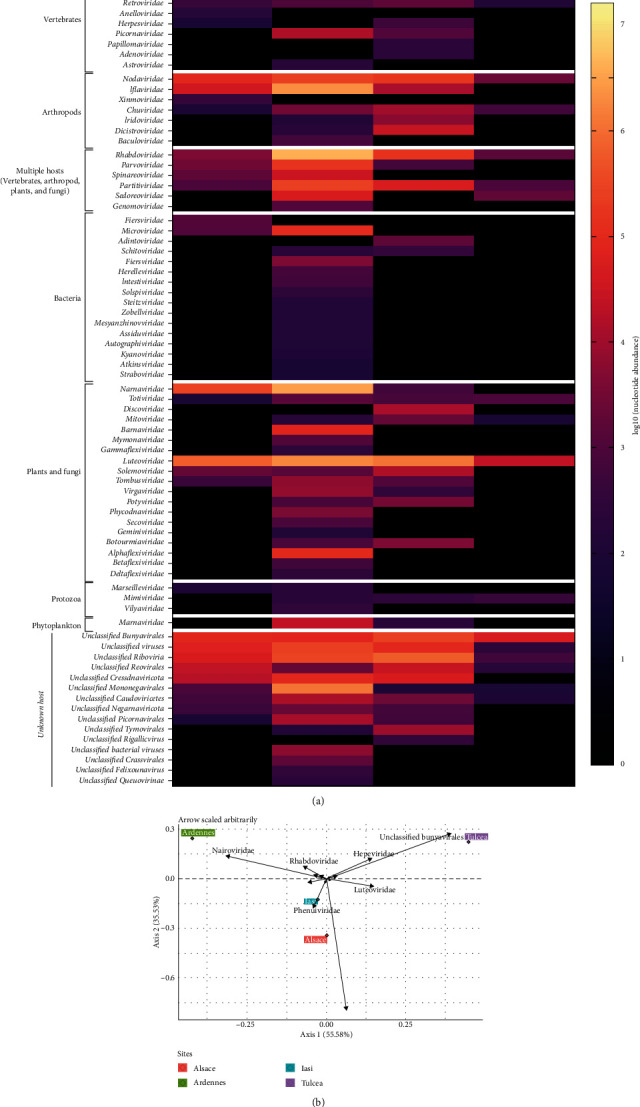
(a) Heatmap representation of the main viral families detected in *I. ricinus* ticks. The rows represent the viral families and the columns are the sites of collection. (b) Principal coordinates analysis (PCoA) of viral families detected in the four *I. ricinus* datasets.

The classified viral reads were distributed into 82 families for which the viral abundance varies considerably, depending on the localization of tick sampling. The highest diversity was observed in ticks from Ardennes region (72 viral families detected), while the lowest abundance occurred in ticks from Tulcea region (21 viral families detected) (Figure 2(a)). However, this result may be due to the different number of ticks collected per site. Some families such as *Flaviviridae*, *Phenuivirdae*, or *Rhabdoviridae* were identified in all sites but with variable abundance while others such as *Sedoreoviridae* or *Orthomyxoviridae* were restricted to a specific location. This suggests the existence of a subset of viral families (such as *Phenuiviridae*, *Nairoviridae*, *Peribunyaviridae*, or *Flaviviridae*) that forms in part the core virome of *I. ricinus*, and the presence of specific families (such as the *Orthomyxoviridae* in Iasi) likely being linked to the acquisition of the virus through blood feeding on a viremic vertebrate host.

For a better overview of specific patterns of viral communities, we performed a principal coordinates analysis (PCoA), starting with the abundance of viral families detected in the four *I. ricinus* datasets (Figure 2(b)). The PCoA revealed a significant difference between the viral communities infecting *I. ricinus* sampled in different biotopes. In Alsace region, the most predominant families were represented by *Flaviviridae* and *Luteoviridae* while, in Ardennes region, the most abundant viral families were *Nairoviridae* and *Rhabdoviridae*. In Tulcea region, most viral sequences belonged to unclassified *Bunyavirales* and *Hepeviridae* families while the *Phenuiviridae* family was dominant in Iasi. These results may suggest that the collection site is a major factor influencing the composition of tick virome.

Among the core virome, apart from sequences belonging to the *Phenuiviridae* or *Nairoviridae* families (see below), sequences related to the *Peribunyaviridae* family mapped onto six novel strains of Bronnoya-like virus (BroBV-like) which were present in all tick pools [22]. BroBV virus was detected in Norway [19], Croatia [24], and Romania [22] and has been described so far only in *I. ricinus* ticks. The *Flaviviridae* family was detected in all libraries but was the most abundant in ticks from Alsace region due to the presence of numerous Jingmen tick virus (JMTV)-related reads. JMTV is a segmented *Flaviviridae*-related virus previously detected in ticks and mammals (including humans, in which it causes febrile illness) from China, Africa, South America, Caribbean, and Europe [37]. Few sequences were related to *Hepeviridae* family and mapped onto Sichuan tick hepe-like virus, sharing 88% amino-acid identity with its closest viral relative, first detected in engorged ticks collected on giant pandas [38]. Sichuan tick hepe-like virus presented a horizontal genome coverage ranging from 52% (in Alsace region) to 100% (in Ardennes region). The unclassified *Reovirales* sequences were detected in two libraries from Alsace and Iasi regions and were assigned to Zoersel tick virus (ZTV), previously identified in *I. ricinus* ticks. ZTV presented lower genome coverage, ranging from 20% in ticks from Alsace to 30% in ticks from Iasi region, and more than 99% amino-acid identity with its closest viral relative from Belgium [39].

In addition to viruses that seem to constitute the core virome of *I. ricinus*, we detected several viruses restricted to a specific location. For example, *Dermacentor reticulatus* pestivirus-like virus 1, a virus related to the *Flaviviridae* family, was restricted to Iasi ticks. The virus presented 94% amino-acid identity with Bole tick virus 4 and a horizontal genome coverage of 100%. These viruses have been associated with ticks feeding on ruminants suggesting that the detection of such viruses in *I. ricinus* ticks from Iasi may reflect the blood meal of the ticks. Similarly, *Sedoreoviridae*-related sequences were restricted to ticks from Tulcea. Also, the complete genome of Chimay rhabdovirus within the *Rhabdoviridae* family, a virus previously identified in *I. ricinus* ticks from Belgium was obtained uniquely from Ardennes ticks.

Finally, within the viral families detected in our study, some of these deserve special mention (such as *Phenuiviridae*, *Nairoviridae*, and *Sedoreoviridae* families) because of their abundance or because they are known or suspected pathogens for animals and humans, being deeply characterized below.

### 3.2. Genetic Characterization of Tribec Virus from Danube Delta, Tulcea

Among the six different genera included in the recently reorganized *Sedoreoviridae* family, one of the most important in terms of spillover potential is the *Orbivirus* genus. In our study, sequences belonging to the *Orbivirus* genus were assigned to Tribec virus (TRBV). The genome of Romanian TRBV was obtained directly from the sequencing reads and derived from one pool containing 64 *I. ricinus* ticks collected from Tulcea region/Danube Delta Reserve. Romanian TRBV shared the same specific genomic structure as other orbiviruses and comprised 10 ORFs coding for the 10 proteins of the virus (Table 1). In all genes, Romanian TRBV was closer to the different strains of TRBV compared to other viruses present in the Great Island group, except in the NS3 protein where it was closer to Muko virus (MKV). The most conserved genes were the VP1 and VP3 while the VP6 and the NS2 presented the highest degree of divergence.

**Figure 3:**
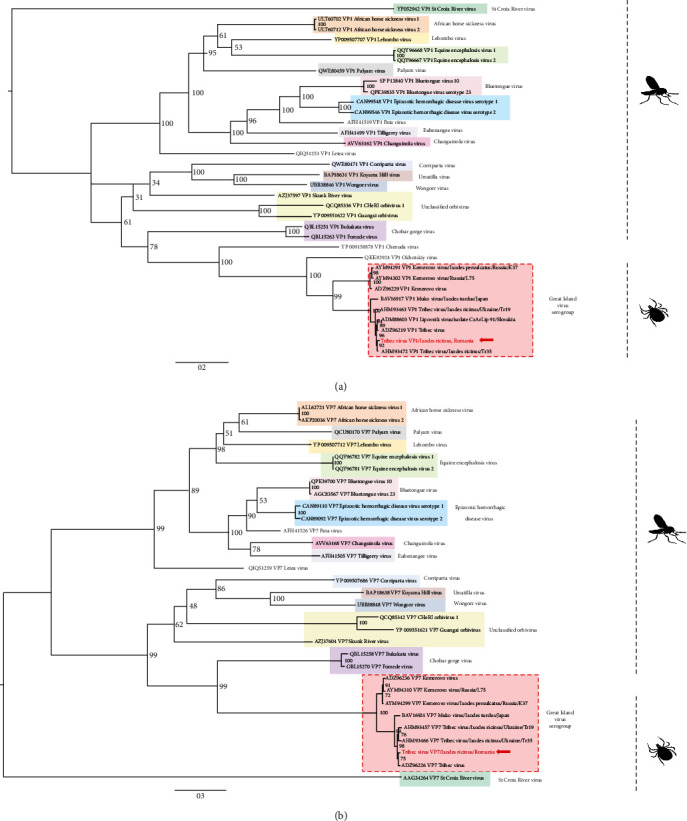
Phylogenetic analysis of Romanian TRBV RdRp and VP7 genes with other viruses within the *Orbivirus* genus: (a) RdRp and (b) VP7 inner capsid.

Phylogenetic analyses performed on the 10 segments confirmed that Romanian TRBV belongs to the *Orbivirus* genus and was positioned in the GIV serogroup, which comprise tick-borne orbiviruses distributed across different geographical areas (Figure 3 and *Supplementary 2*). The Romanian TRBV generally clustered in a specific clade, distinct from Kemerovo virus, that comprises other TRBV isolates but with few variations when considering the different segments: in VP1 and VP6 segments, TRBV/Romania clustered in a subclade with TRBV-Tr35/Ukraine while being clustered with the prototype strain in VP2, VP7, and NS3 genes, and with TRBV-Tr19/Ukraine in the VP5 gene. In NS1, TRBV/Romania placed at the root of the reliably supported group of TRBV-Tr19/Ukraine, TRBV-Tr35/Ukraine, TRBV/prototype, and MUV/Japan, while in NS2 and VP4, Muko virus was placed at the root of the subclade formed by TRBV/Romania, TRBV-Tr19/Ukraine, TRBV-Tr35/Ukraine, and TRBV/prototype. The Romanian TRBV strain exhibits distinct clustering patterns depending on the segment, indicating it underwent diverse evolutionary pathways during its circulation through a vertebrate-arthropod infection cycle and suggesting a possible reassortment at the origin of Romanian TRBV.

### 3.3. Genetic Characterization of *Phenuiviridae* Viruses

*Phlebovirus* is the unique viral genus of the *Phenuiviridae* family that is able to infect vertebrates, including humans through a wide range of arthropod vectors (sandflies, mosquitos, or ticks). Tick-transmitted phleboviruses are clustered into four serogroups: Bhanja virus (BHAV) and Bhanja-related viruses responsible for febrile symptoms; severe fever with thrombocytopenia syndrome virus (SFTSV); member of SFTSV serogroup and related to hemorrhagic fever illnesses in Asia; and Uukuniemi virus (UUKV) and Kaisodi virus (KSOV) in the Uukuniemi/Kaisodi serogroups that have not been recognized as human pathogens [40–42]. The genome of these viruses is constituted of three segments: the L segment encodes for the viral RdRp, the M segment encodes for the glycoprotein precursor that will subsequently matured into two glycoproteins (Gn and Gc) and a nonstructural protein (NS), and the S segment which encodes the nucleoprotein (NP) and a nonstructural NS protein.

**Figure 4:**
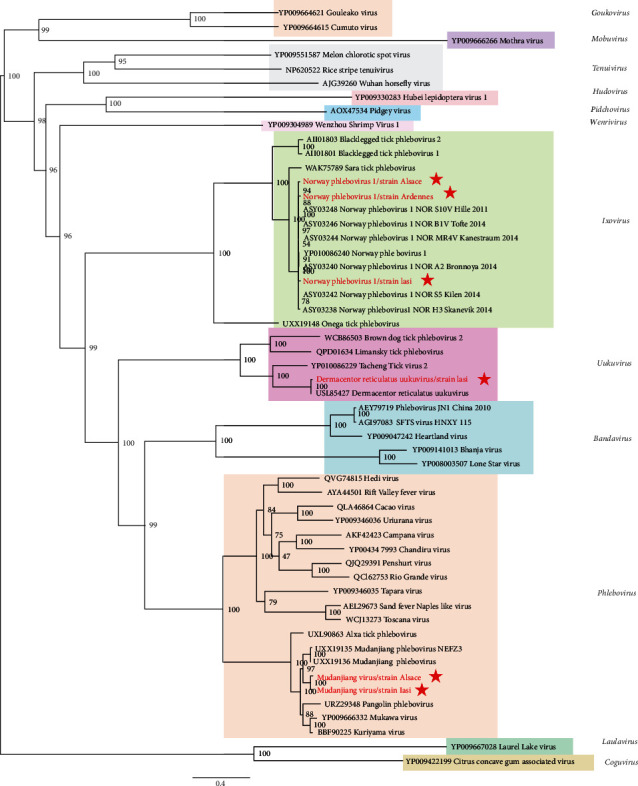
Phylogenetic analysis of Romanian MDJV, NWPV1, and DRUV1 with other viruses within the *Phenuiviridae* family. MDJV, Mudanjiang virus; NWPV1, Norway phlebovirus 1; DRUV1, *Dermacentor reticulatus* uukuvirus 1.

In the current study, we detected two viruses belonging to the *Phlebovirus* genus and related to Mudanjiang virus (MDJV). MDJV-like/Alsace and MDJV-like/Iasi presented the same trisegmented genome architecture specific to phleboviruses. Comparison of the RdRp sequences of Alsace and Iasi MDJV strains demonstrated a 99% amino-acid identity between each other, and a lower (around 90%) degree of conservation with the two strains of MDJV identified in *Ixodes persulcatus* in China [43]. MDJV-like/Alsace and MDJV-like/Iasi were more distantly related to Mukawa virus (YP009666332), Kuriyama virus (BBF90225), Pangolin phlebovirus (URZ29348), and Alxa phlebovirus (UXL90863), with lower amino-acid identities ranging from 86.86% to 83.11% in the RdRp. Phylogenetic relationships between the Romanian and French strains of Mudanjiang virus and other phleboviruses showed that our strains clustered in a clade that comprises phleboviruses transmitted by ticks recently detected in *I. persulcatus* and *H. concinna* from China (Mudanjiang virus) [43, 44] and Japan (Mukawa and Kuriyama virus) [45], respectively, except for Pangolin phlebovirus for which the vector is yet unknown. This clade is rooted by Alxa phlebovirus, a tick-borne phlebovirus detected in Chinese tick metagenomes in 2016 and 2019, forming a distinct but well-supported clade within the *Phlebovirus* genus (Figure 4).

We also identified sequences related to a novel *Uukuvirus*, named *Dermacentor reticulatus* uukuvirus 1-like (DRUV1), in *I. ricinus* ticks from Iasi, Romania. DRUV1 presented two segments encoding for the RdRp and the nucleoprotein and a horizontal genome coverage of 99.8% and 31.3%, respectively. Phylogenetic analysis showed that Romanian DRUV1 clustered together with DRUV1 (USL85427) identified in Croatia in 2022 [24], but placed close to Tacheng tick virus 2, previously associated with human infections in China [36] (Figure 4).

Among the *Ixovirus* genus, we detected three Norway phlebovirus 1 (NWPV1)-like strains in *I. ricinus* ticks from Alsace, Ardennes, and Iasi, with a horizontal genome coverage ranging from 90% to 100% for the L segment and from 61% to 100% for the S segment. Phylogenetic analysis performed on the RdRp confirmed that the three NWPV1 strains clustered within the *Ixovirus* genus, with other NWPV1 identified previously in *I. ricinus* from Norway [19] (Figure 4).

### 3.4. Genetic Characterization of the Nairoviridae Family

Of major interest is the *Orthonairovirus* genus which is transmitted mainly by ticks and includes some of the most significant tick-borne pathogens for public health [46]. The genome of orthonairoviruses is usually trisegmented: the L segment coding for the RNA-dependent RNA polymerase (RdRp), the M segment encoding for the glycoprotein (Gn and Gc) and the nonstructural protein NSm, and the S segment encoding for the nucleocapsid (NP) and the nonstructural protein NSs. However, recent studies have revealed the presence of a new group of bisegmented orthonairoviruses, apparently missing the M segment [19, 30].

**Figure 5:**
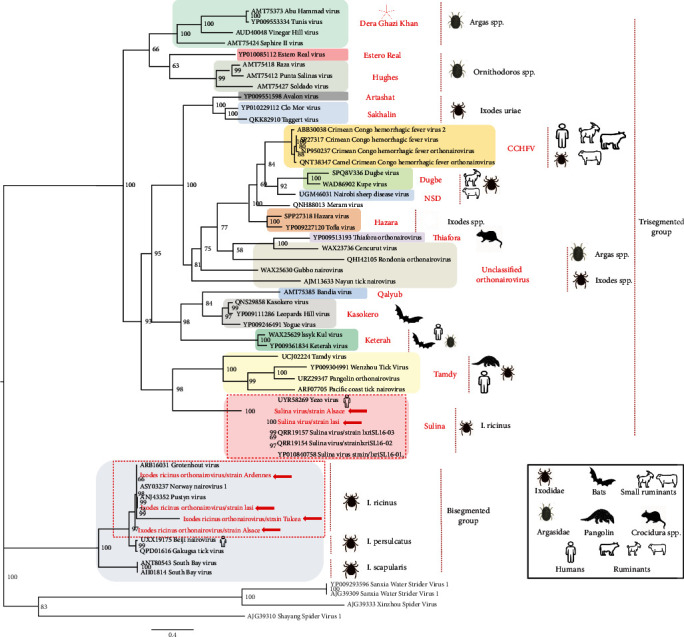
Phylogenetic analysis of Romanian IRNV and SULV strains with other viruses within Nairoviridae family. IRNV, *Ixodes ricinus* orthonairovirus; SULV, Sulina virus.

Here, we identified four bisegmented strains, provisionally named *Ixodes ricinus* orthonairovirus (IRNV) detected both in Romanian (Iasi and Tulcea) and French (Alsace and Ardennes) *I. ricinus* ticks. The pairwise comparison of our strains with representative bisegmented *Orthonairovirus* strains performed on the NP gene showed a high degree of amino-acid conservation with Norway nairovirus 1, Pustyn virus, and Grotenhout virus, ranging from 100% to 98%; but lower identities were observed for Beiji virus and Gakugsa virus, ranging from 86.41% to 84.78%. South Bay virus exhibited a more distant profile than other viruses, with 56.96% and 57.69% of amino-acid identity, respectively. Phylogenetic reconstruction placed *Ixodes ricinus* orthonairovirus strains in a clade comprising novel bisegmented nairoviruses associated with *I. ricinus* ticks (namely Norway nairovirus 1, Pustyn virus, and Grotenhout virus) and in a sister subclade of Beiji nairovirus, a newly identified human pathogen [30] (Figure 5).

Additionally to the group of bisegmented nairoviruses, we detected in *I. ricinus* ticks from Alsace and Iasi regions, two novel trisegmented strains close to Sulina virus (SULV), first identified in *I. ricinus* ticks from Danube Delta, Romania [47]. Our SULV strains presented an amino-acid identity ranging from 84% to 99% and a horizontal genome coverage ranging from 42% to 89% depending on the segment and strain considered. SULV sequences were also detected in ticks from Ardennes region, but due to the low genome coverage, they were excluded from the analysis. Phylogenetic analysis performed on the NP gene confirmed that the two strains of SULV identified in our study belong to the trisegmented group of nairoviruses and clustered in the Sulina genogroup comprising other SULV strains originating from Romania and Yezo virus. Interestingly, this serogroup is currently associated to the *Ixodes* spp. vector, with *I. persulcatus* transmitting Yezo virus to humans in Asia [31] (Figure 5). The ability of *I. ricinus* to transmit-related viruses of this serogroup to vertebrates has therefore to be evaluated.

### 3.5. Determination of the Arbovirus Potential of *Ixodes ricinus* Viruses

**Figure 6:**
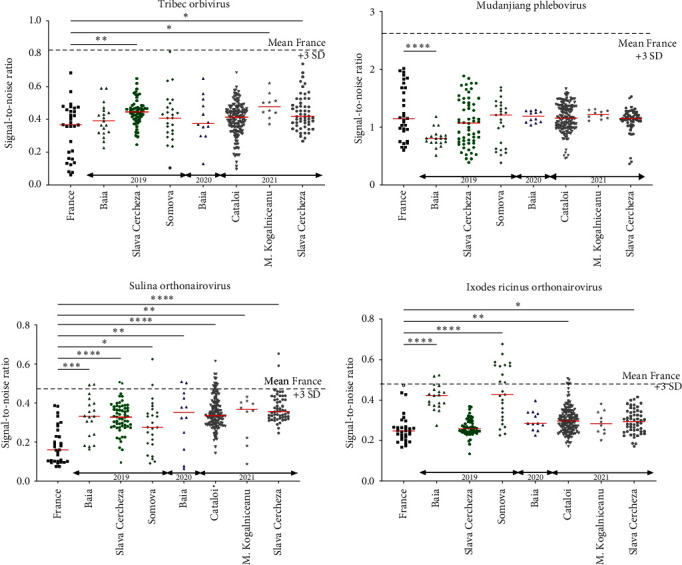
Results of the luciferase immunoprecipitation (LIPS) small-ruminant screening against Romanian TRBV, SULV, IRNV, and MDJV. French sheep sera (black) served as likely nonexposed negative controls. Sera were collected in 2019 (green), 2020 (blue), and 2021 (gray) in different localities represented by different dots. The ANOVA nonparametric Kruskal–Wallis test was conducted to compare each subpopulation to the reference French population. Only significant differences are presented and labeled as  ^*∗*^,  ^*∗*^ ^*∗*^, and  ^*∗*^ ^*∗*^ ^*∗*^ ^*∗*^ according to the level of significance.

To test if one or more viruses identified among *I. ricinus* ticks collected on small ruminants are able to infect their vertebrate hosts and, therefore, could constitute putative novel tick-borne arboviruses, we developed LIPS-based serological screening focusing on the VP7-inner capsid of Tribec orbivirus, the glycoprotein of Mudanjiang phlebovirus, and the nucleoprotein of Sulina and *Ixodes ricinus* orthonairoviruses. In the absence of any positive control serum, we chose a positivity threshold defined as the mean of signal-to-noise ratio of nonexposed French ruminant sera plus three standard deviations. Results are presented in Figure 6. No Romanian sheep or goat serum tested positive for TRBV and MDJV. This negative result indicates that the maximal seroprevalence is 0.9% (*p*=0.05). However, 25 sera collected from Baia (2019 and 2020), Slava Cercheza (2019 and 2021), Somova (2019), and Cataloi (2021) slightly exceeded the positivity threshold for SULV antigen. Similarly, for IRNV antigen, 16 sera collected from Somova and Baia (2019) presented a luciferase activity higher than the positivity threshold. More importantly, the median level of antibody response of Baia-2019 and Somova-2019 sera were significantly higher compared to the French nonexposed sera, suggesting that the Romanian small ruminants may have been exposed to *Ixodes ricinus* orthonairovirus. Interestingly, in Somova-2019, all sera exceeding the putative positivity threshold were collected on sheep while goat sera were all negative.

## 4. Discussion

In this study conducted as part of a global research effort to understand the virome of *I. ricinus* ticks from two European countries (France and Romania), we combined virome characterization with comprehensive phylogenetic analyses to investigate viruses that are closely related to tick-borne arboviruses. We further performed a sero-epidemiological study in the Romanian small ruminant populations from Danube Delta in order to characterize the arbovirus potential of candidate viruses. If a positive finding suggests the ability of a candidate tick-associated virus to infect small ruminants, negative findings inform on the probable nontransmitted nature of a given virus and its probable restriction to ticks (within the limits of the vertebrate species and the number of sera tested), increasing therefore the knowledge of the host spectrum of tick-borne viral communities. Such knowledge, usually lacking in many metagenomics studies, is a prerequisite for improving targeted surveillance of emerging tick-borne arboviruses.

Among the viral communities detected in *I. ricinus* ticks, we described novel viruses belonging to the *Phenuivirdae* and *Nairoviridae* families and we reported the first complete genome of a novel strain of Tribec virus, an important pathogen associated with neurological symptoms in humans. Phylogenetic analyses of the 10 viral segments revealed a close association between the Romanian strain and other TRBV strains isolated from Ukraine [11], at the border of Romania. This finding suggests the presence of a continuum of tick populations (and their associated viral communities that include arboviral pathogens) across Eastern Europe which could result, due to the wide distribution of *I. ricinus* in Europe [2] to the silent circulation of TRBV-related viruses in Western and Eastern European countries. In the present study, Tribec virus was detected in engorged *I. ricinus* ticks from the Danube Delta, which constitutes one of the most important wetland areas for migratory birds and a major hub for bird migration from Africa and Asia [48]. Migratory birds are able to travel over long distances between their breeding site and their wintering site, carrying with them ectoparasites, including ticks and associated microorganisms, which may suggest the presence of TRBV along their migratory routes, possibly leading to a high risk of introduction of the virus in new areas. In addition, and despite the fact that previous studies [11] mentioned *I. ricinus* ticks as the main vector of TRBV, the virus has been detected in other tick species, including *Haemaphysalis punctata* ticks from the western part of Romania [49]. This observation highlights the low degree of vector restriction of TRBV, which can also favor its dissemination over distant areas and its endemization in new areas that are not colonized by *I. ricinus*.

Even if rodents and small ruminants are considered as the main reservoirs of TRBV [13, 16], animal diseases caused by Tribec virus infection have not been reported yet. Few studies indicated that the seroconversion has been present in birds, but their role in the transmission of the virus remains unclear [15]. In our study, small ruminant sera were tested and none of them exceeded the positivity threshold, suggesting that infection of small ruminants by TRBV in the Danube Delta, if they occur, is rare. To obtain a comprehensive understanding of the host spectrum and get primary insights into the ecological cycle of Romanian TRBV, it is crucial to conduct further investigations, such as exploring a larger number of vertebrate hosts, including rodents and birds, which represent some of the most numerous vertebrate species present in the Delta.

The family *Phenuiviridae* consists of segmented negative-strand ssRNA viruses, encompassing the genera *Goukovirus* and *Phasivirus* (viruses specific to insects), *Tenuivirus* (viruses that infect plants), and *Phlebovirus* (viruses that infect animals). *Ixodidae* ticks serve as the primary vectors for tick-borne phleboviruses [50]. Birds and small mammals have been proposed as reservoirs for these viruses, while humans and domestic animals are considered accidental hosts [51, 52]. Our study identified two strains of Mudanjiang virus (MDJV) phylogenetically related to MDJV tick-borne isolates from Asia [53]. Interestingly, MDJV strains formed two distinct subclades. One subclade consisted of MDJV strains detected in China [53], specifically in *I. persulcatus* ticks, and the other subclade comprised MDJV strains from Alsace/Iasi, identified in *I. ricinus* ticks. Furthermore, the phylogenetic analysis revealed that the Romanian and French strains of MDJV were closely related to the Pangolin MDJV strain detected in China, in 2018. This suggests that these viruses could potentially infect vertebrates, particularly in regions where vectors, reservoirs, and animals coexist. In contrast, our serological research indicated that MDJV does not appear to have the ability to infect small ruminants, or at very low prevalence. Therefore, we cannot exclude that this virus could constitute tick endosymbionts that represent viral ancestors of the known trisegmented tick-borne phleboviruses, as previously suggested [43].

In the same *Phenuiviridae* family, we identified *Dermacentor reticulatus* uukuvirus 1 and Norway phlebovirus 1 that clustered in *Uukuvirus* and *Ixovirus* genera, respectively. So far, these viruses were only identified in ticks [24], so their potential ability to infect multiple hosts is questionable. Indeed, the phylogenetic proximity between *Dermacentor reticulatus* uukuvirus 1 and a human clinical isolate from Northwestern China [36], which has a history of tick bite, raises concerns regarding the potential for Romanian ticks to harbor novel arboviruses, and highlights the need for further investigation and surveillance to better understand the diversity and potential public health implications of *Phenuiviridae* arboviruses in Romanian ticks.

The detection of viruses belonging to the *Nairoviridae* family in the virome of *I. ricinus* ticks is also a matter of concern. Many members of the *Orthonairovirus* genus are known to be transmitted by ticks to mammalian hosts such as bats, rodents, and ungulates, and infections are typically asymptomatic [54]. Here, we successfully identified and characterized four distinct strains of a newly discovered bisegmented nairovirus, tentatively named *Ixodes ricinus* orthonairovirus (IRNV). These strains are placed within a clade that includes recently discovered nairoviruses that appear to be predominantly detected in *I. ricinus* ticks from Northern Europe (Belgium and Norway) [19, 32] and which constitutes a sister clade of a group of novel bisegmented tick-borne pathogens associated with human febrile illness in China [30]. The ability of *Ixodes ricinus* orthonairovirus, and viruses of the same clade, to infect vertebrates is therefore questionable. Similar observations can be considered for the two strains of the trisegmented Sulina virus (SULV), as they clustered together in the same clade as Yezo virus [31] detected in a tick-bitten patient. These viruses are phylogenetically close to the Tamdy genogroup, which comprises tick-borne human pathogens such as Tamdy virus or Tacheng tick virus 1 [55, 56]. Our serological surveys show that part of the small ruminant population from Danube Delta could have been exposed to viruses related to these bi- or tri-segmented nairoviruses. Indeed, some paradoxal serological studies on the circulation of Crimean–Congo hemorrhagic fever virus (CCHFV) conducted in the same investigated area are in favor of this hypothesis [57]. While the virus was never detected in tick populations, sero-epidemiological studies revealed a puzzling high prevalence ranging from 27.8% to 74%, when looking at IgG antibodies directed against the nucleoprotein of CCHFV [57]. In addition, no human cases of CCHFV infection have been reported thus far in Romania. To investigate if the apparent high seroprevalence of CCHFV in small ruminants could be attributed to the presence of another related virus currently circulating in the region, we determined if the prevalence and the level of antibody response against bi- or tri-segmented nairoviruses detected in *I. ricinus* ticks were higher in CCHFV-positive sera compared to CCHFV-negative sera (*Supplementary 3*). No significant differences were observed between the two groups of ruminant sera, but the few sera that exceeded the proposed positivity threshold for *Ixodes ricinus* orthonairovirus belonged all to sheep, while goat sera exhibited a lower antibody response. This low level of positivity might be due to the presence of cross-reactive antibodies with related nairoviruses because the nucleoprotein, which presents lower specificity than the glycoprotein, is targeted. Therefore, more-specific serological tests such as seroneutralization are needed to confirm this observation, but this would require the isolation of the virus in high biosecurity laboratories.

## 5. Conclusions

In conclusion, the identification of novel viruses plays a crucial role to monitor silent viral emergences. Equally important is the need to determine among the vast number of viruses harbored in vectors and reservoirs, which one is able or not to cross the species and to understand why certain viruses, which share a close phylogenetic relationship with zoonotic viruses, remain incapable of crossing the species barrier.

## Figures and Tables

**Table 1:** Amino-acid identities on the 10 TRBV segments within Great Island virus serogroup.

			Amino-acid identity %						
Segment	Length LIAA ORF	TRBV/Tr35 (%)	TRBV/Tr19 (%)	TRBV/prototype (%)	KMV/L75 (%)	KMV/prototype (%)	KMV/K37 (%)	MKV (%)	PV
VP1	1,286	98.75	98.52	98.75	78.43	78.74	78.19	94.54	98.52%
VP2	554	94.38	94.20	94.57	82.61	82.25	81.70	58.70	N/A
VP3	908	99.78	99.56	99.78	90.97	91.19	91.19	98.68	N/A
VP4	628	99.52	97.42	97.58	75.32	76.61	77.10	91.29	N/A
VP5	537	98.21	98.41	99.40	87.45	87.45	87.65	93.23	94.61%
VP6	312	91.03	87.82	82.05	61.51	61.51	62.46	77.24	N/A
VP7	357	98.02	97.18	98.59	85.31	85.88	86.16	95.35	N/A
NS1	529	96.21	95.83	93.94	66.10	65.72	65.53	90.53	N/A
NS2	368	97.28	97.28	97.55	63.24	63.51	63.24	88.59	N/A
NS3	214	78.10	80.95	91.90	77.62	77.14	77.14	93.81	N/A

TRBV, Tribec virus; KMV, Kemerovo virus; MKV, Muko virus; LIPV, Lipovnik virus.

## Data Availability

Sequence data reported in this manuscript were submitted to GenBank (http://www.ncbi.nlm.nih.gov/Genbank) under the accession numbers OR645553–OR645574 and OR613114–OR613127. The raw data have been deposited in SRA database under the BioProject ID PRJNA1018895.

## Supplementary Materials

Supplementary 1 Study sites, tick sampling, libraries preparation, small ruminant sera collection, and bioinformatic analysis.

Supplementary 2 Phylogenetic analysis on the amino-acid sequences of the 10 segments of Great Island serogroup.

Supplementary 3 Prevalence and antibodies level response against bi- and tri-segmented nairoviruses between CCHFV-positive sera and CCHFV-negative sera.
