# Supplementary material for: Diversity of Viruses in Ixodes ricinus in Europe including Novel and Potential Arboviruses
Source: Transbound Emerg Dis. 2023 Nov 21;2023:6661723. doi: 10.1155/2023/6661723 (PMC12017061; doi:10.1155/2023/6661723)
Supplement: Supplementary 1 — Study sites, tick sampling, libraries preparation, small ruminant sera collection, and bioinformatic analysis. [file 6661723.f1.pdf]

Supplementary materials

Study sites, tick sampling and libraries preparation

Ticks collected in Romania, Danube Delta Reserve, 2020–2021

A number of 202 ticks were directly picked from adult sheep in Slava Rusa village, Danube Delta region, Romania in October 2020 and May 2021. The collected ticks were divided into 20 pools before extraction (three to fifteen ticks per pool). Total RNA was extracted from crushed specimens using the Maxwell® RSC simplyRNA Tissue Kit or the TRIzol Reagent (Invitrogen), followed by the RNeasy mini kit (Qiagen), in accordance with the manufacturer's instructions. The 20 pool extract ticks were combined to form five NGS libraries using the SMARTer Stranded Total RNA-seq kit v3-Pico input mammalian (Clontech, TaKaRa Bio, San Jose, CA, USA) or the NEBNext Ultra II DNA Library Prep Kit (New England Biolabs, Evry, France). The NGS libraries were validated using an Agilent Bioanalyzer and the quantification was conducted using an Invitrogen Qubit 2.0 Fluorometer (Carlsbad, CA, USA). Libraries were sequenced on either an Illumina NextSeq500 Sequencer in a single-read 1 × 150 bp format or sequenced using an Illumina Nextseq2000 Sequencer (Illumina Inc., Saint Diego, CA, USA) in a 2 × 100 bp format.

Ticks collected in Romania, Iasi County, 2015

A total of 315 ticks were collected by flagging method between March and September 2015. Ticks were sampled from six collection sites (Bucium, C.A. Rosetti, Breazu, Ciric, Cetăţuia and Bârnova) representing suburban sites intended for recreational activities in Iasi County, Romania. Ticks were washed to remove any external contaminant, and total RNA was extracted from individual ticks using the Nucleospin RNA II kit (Macherey-Nagel, Duren, Germany) following the manufacturer's recommendations. Tick RNA was pooled before sequencing to form three pools that were processed by the REPLI-g® WTA kit (Qiagen, Hilden, Germany). The libraries were sequenced using paired-ends 2 × 150 bp format and were outsourced to Integragen Company (Evry, France).

Ticks Collected in France (Alsace and Ardennes regions), 2010–2012

A number of 2236 questing nymph and adult ticks were sampled from the environment, in northeastern France, between 2010 (Alsace) and 2012 (Ardennes) and processed as previously described. Ticks were washed to remove external contaminants. The nymphs were grouped into pools of 15 specimens (116 pools in total) and adults were treated individually. Ticks were crushed into Dulbecco's modified Eagle's medium, supplemented with 10% fetal calf serum, before a total RNA extraction with the Nucleospin RNA II kit (Macherey-Nagel, Duren, Germany). The RNA samples were pooled according to their geographical location and reverse-transcribed into cDNA using the SuperScript III Reverse Transcriptase and random hexamers (Invitrogen, Carlsbad, CA, USA). The tick material was then randomly amplified using the multiple displacement amplification (MDA) protocol with phi29 polymerase and random hexamers as described previously. Library preparations and sequencing with an Illumina HiSeq2000 (Illumina Inc., Saint Diego, CA, USA) sequencer on a 2 × 100 bp format were outsourced to DNAVision Company (Charleroi, Belgium).

Determination of Tick Species

Ticks were initially identified under a stereomicroscope using standard morphological keys and the tick species was further confirmed using the Barcode of Life Data Systems (BOLD). Briefly, all trimmed reads were mapped onto the Ixodidae Cytochrome Oxydase sub-unit I (COI) sequences included in the BOLD database. Mapped reads were de novo assembled, and the resulting contigs were uploaded to the BOLD Identification System (https://www.boldsystems.org/index.php/IDS_OpenIdEngine) for species identification. Results were finally validated by BlastN. All ticks analyzed in the present study were identified as *Ixodes ricinus*.

Virus Assignation

After sequencing, raw reads were processed with an in-house bioinformatics pipeline called Microseek, which comprises a quality check and the trimming of raw reads followed by read normalization prior to assembly into contigs and the generation of non-assembled singletons. ORF predictions of both contigs and singletons were performed, and a taxonomic assignation of protein sequences was performed by Blast-based querying using three successive, specialized RVDBs, followed by generalist (NCBI/nr and NCBI/nt) databases.

Principal Coordinates analysis

Tables and figures describing viral families relative abundances were used for statistical analyses (Principal Coordinates Analyses) in R software (v4.2.1) using ade4 and Vegan packages, and plots were generated with ggplot2 package. Principal Coordinates Analysis (PCoA) conducted on normalized counts to visualize differences between sites. The first two axis capturing 91.11 % of variability within our data are represented.

Phylogenetic Analyses

Phylogenetic analyses were performed on the following genes: the conserved non-structural RNA-dependent RNA polymerase (RdRP), the nucleoprotein (NP), and the VP2-VP7 inner capsids. The MAFFT (Multiple Alignment using Fast Fourier Transform) aligner was used to align the complete ORFs with other representative sequences of viral orders/families under the L-INS-i or G-INS-I parameters. The best amino-acids substitution models that fitted the data were determined with ATGC Smart Model Selection implemented in http://www.atgc-montpellier.fr/phyml-sms/ using the corrected Akaike information criterion. These were LG+G for all phylogenies. Based on the selected substitution model, phylogenetic trees were generated using the maximum likelihood (ML) reconstruction method provided through the IQ-TREE program (http://iqtree.cibiv.univie.ac.at/). The nodal support was assessed using the approximate Bayes parameter. To generate identity matrices for all the analyzed virus genomes, amino-acid sequences were aligned with MAFFT with the same parameters as those used for phylogenetic reconstructions, and matrices were generated using CLC Main Workbench v.21.0.4 (Qiagen, Hilden, Germany).

Small ruminant sera collection

A total of 331 blood samples were collected from small ruminants between 2019 and 2021, from six different villages from Tulcea County, Danube Delta region, Romania. Details regarding the

number of serum samples and the collection sites are presented in Table S1. These sera were collected with the owner’s approval, by a local veterinarian from Tulcea County. Additionally, as a control group representing non-exposed individuals, 31 sera from healthy sheep (kindly provided by Dr. Stephan Zientara and Emmanuel Breard, Anses, Maisons-Alfort, France) collected in a distinct location in France, separate from the collection site of the French ticks, were included in the study. All samples were shipped to the Institut Pasteur in Paris, France, for further analysis.

Table S1. Ruminant serum samples collected from Tulcea County, Romania

**Year of collection**	**Collection site**	**Species of small ruminants**	**Number of serum samples**
**2019**	Baia	Sheep	20
	Cataloi	Goat	27
		Sheep	29
	Slava Cercheză	Sheep	58
	Somova	Sheep	25
**2020**	Baia	Sheep	6
		Goats	6
**2021**	Cataloi	Sheep	96
	Mihail Kogalniceanu	Sheep	10
	Slava Cercheză	Sheep	54
	**TOTAL**		**331**

Serological screening of small ruminants

Choice of antigens

To maximize the probability of detecting antibodies specific to the viruses detected in ticks, we targeted three proteins that we considered should be immunogenic: VP7 from Tribec orbivirus and the glycoprotein Gn from Mudanjiang phlebovirus, which are exposed on the viral surface and therefore accessible to the immune system, and the nucleoprotein of Sulina and Ixodes ricinus orthonairoviruses, To design the antigens, as there is currently no experimental structures available for any of these proteins, we use alphafold2 (AF2) (PMID: 34265844) to predict their structures. To assess the quality of the models produced, AF2 produced a confidence metric termed pLDTT, on a scale from 0 to 100. Values of pLDTT higher than 70 reflect reliable models with correct backbone prediction. As the VP7 of other orbiviruses (PMID: 7816101), the AF2 model for VP7 from Tribec virus (pLDTT = 90) folds into two domains, a alpha-helical domain that interacts with VP3 and beta-structured domain (residues 118-257) that projects on the viral surface, which we have used as antigen. Using the AF2 model of the Gn/Gc complex from Mudanjiang phlebovirus (pLDTT = 77), we identified the two sub-domains of the ectodomain of Gn (PMID: 34960636): head (aa. 31-334) and base (aa. 335-444) and the ectodomain of Gc (aa.

517-1025). We decided to use the head of Gn as antigen because this domain turned out to be the most immunogenic in other phleboviruses.

Statistical Tests

Statistical analyses were conducted using GraphPad Prism v.8 (GraphPad Software, San Diego, CA, USA). The signal-to-noise light unit (LU) ratios between each group of sera were compared using the Kruskal–Wallis ANOVA and Dunn's multiple comparisons tests with the French cohort used as a reference.
