# Supplementary figures and images for: Diversity of Viruses in Ixodes ricinus in Europe including Novel and Potential Arboviruses
Source: Transbound Emerg Dis. 2023 Nov 21;2023:6661723. doi: 10.1155/2023/6661723 (PMC12017061; doi:10.1155/2023/6661723)

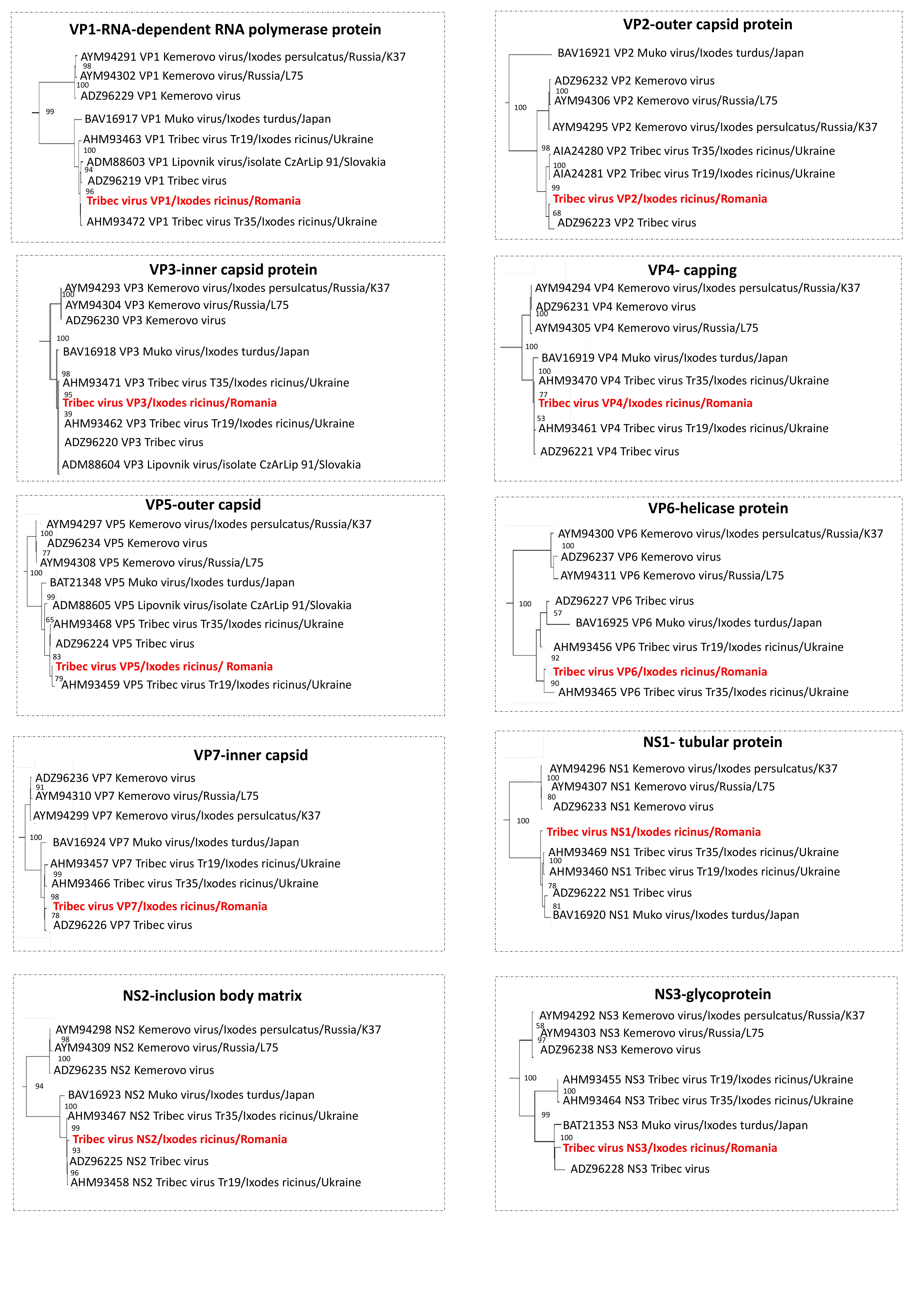

Supplement: Supplementary 2 — Phylogenetic analysis on the amino-acid sequences of the 10 segments of Great Island serogroup. [file 6661723.f2.jpg]

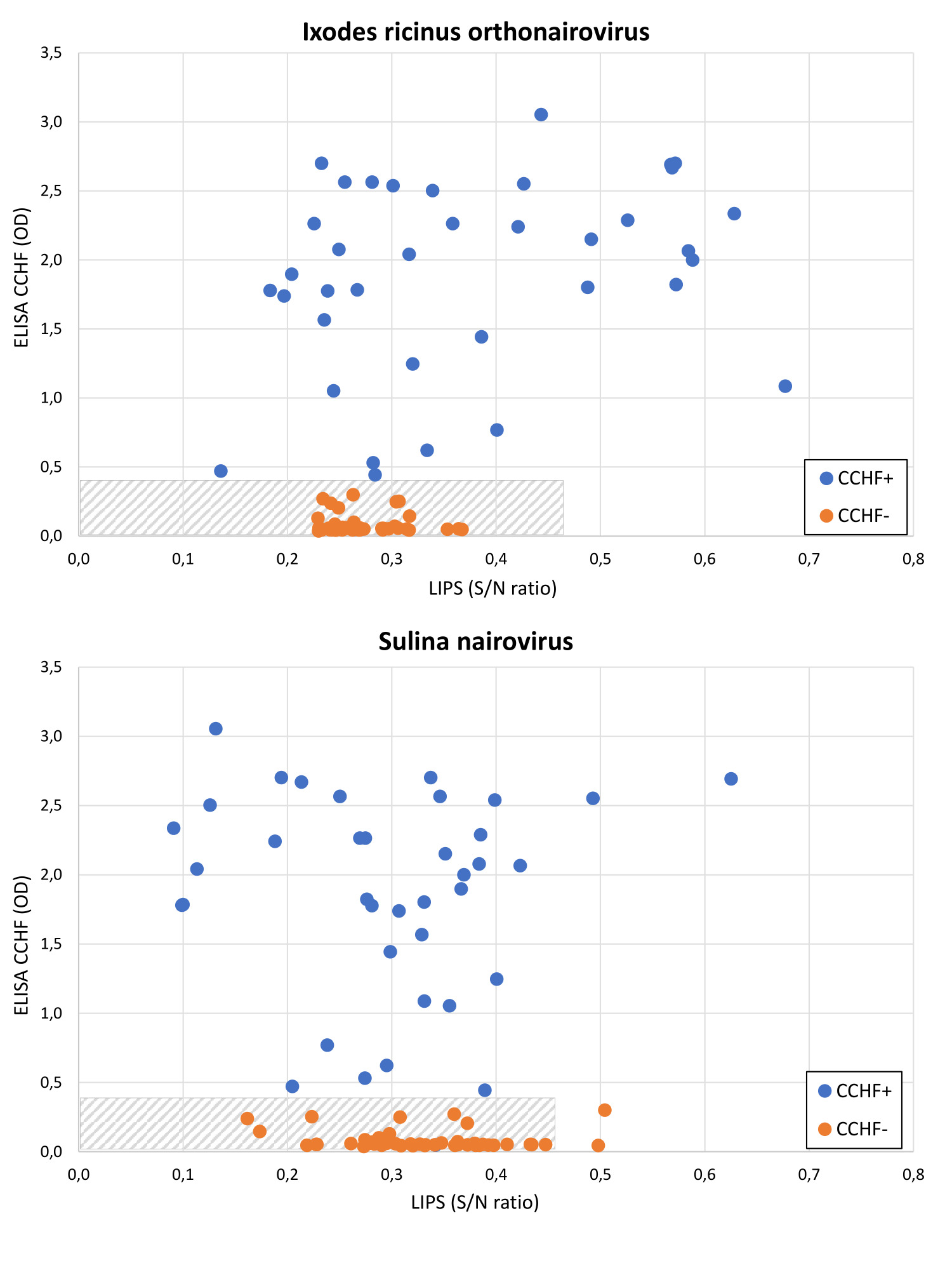

Supplement: Supplementary 3 — Prevalence and antibodies level response against bi- and tri-segmented nairoviruses between CCHFV-positive sera and CCHFV-negative sera. [file 6661723.f3.jpg]
